# Making hard choices easier: a prospective, multicentre study to assess the efficacy of a fertility-related decision aid in young women with early-stage breast cancer

**DOI:** 10.1038/bjc.2012.61

**Published:** 2012-03-13

**Authors:** M Peate, B Meiser, B C Cheah, C Saunders, P Butow, B Thewes, R Hart, K-A Phillips, M Hickey, M Friedlander

**Affiliations:** 1 Prince of Wales Clinical School, University of NSW, Randwick, New South Wales, Australia; 2 Department of Medical Oncology, Prince of Wales Hospital, Randwick, New South Wales, Australia; 3 Neuroscience Research Australia, University of NSW, Randwick, New South Wales, Australia; 4 School of Surgery, University of Western Australia, Crawley, Western Australia, Australia; 5 Centre of Medical Psychology and Evidence Based Decision-Making, The University of Sydney, Sydney, New South Wales, Australia; 6 School of Women's and Infants Health, The University of Western Australia, King Edward Memorial Hospital, Subiaco, Western Australia, Australia; 7 Division of Cancer Medicine, Peter MacCallum Cancer Centre, East Melbourne, Victoria, Australia; 8 Department of Medicine, St Vincent's Hospital, The University of Melbourne, Melbourne, Victoria, Australia; 9 Department of Obstetrics and Gynaecology and the Royal Women's Hospital, The University of Melbourne, Parkville, Victoria, Australia

**Keywords:** breast cancer, fertility, decision-making, young women, decision aid

## Abstract

**Background:**

Fertility is a priority for many young women with breast cancer. Women need to be informed about interventions to retain fertility before chemotherapy so as to make good quality decisions. This study aimed to prospectively evaluate the efficacy of a fertility-related decision aid (DA).

**Methods:**

A total of 120 newly diagnosed early-stage breast cancer patients from 19 Australian oncology clinics, aged 18–40 years and desired future fertility, were assessed on decisional conflict, knowledge, decision regret, and satisfaction about fertility-related treatment decisions. These were measured at baseline, 1 and 12 months, and were examined using linear mixed effects models.

**Results:**

Compared with usual care, women who received the DA had reduced decisional conflict (*β*=−1.51; 95%CI: −2.54 to 0.48; *P*=0.004) and improved knowledge (*β*=0.09; 95%CI: 0.01–0.16; *P*=0.02), after adjusting for education, desire for children and baseline uncertainty. The DA was associated with reduced decisional regret at 1 year (*β*=−3.73; 95%CI: −7.12 to −0.35; *P*=0.031), after adjusting for education. Women who received the DA were more satisfied with the information received on the impact of cancer treatment on fertility (*P*<0.001), fertility options (*P*=0.005), and rated it more helpful (*P*=0.002), than those who received standard care.

**Conclusion:**

These findings support widespread use of this DA shortly after diagnosis (before chemotherapy) among younger breast cancer patients who have not completed their families.

Approximately 3–7% of women with early-stage breast cancer are under 40 years of age at diagnosis (Australian Institute of Health and Welfare (AIHW) and Australasian Association of Cancer Registries (AACR), 2004; Bouchardy *et al*, 2007; Peate *et al*, 2009; American Cancer Society, 2011–2012). Most women receive adjuvant chemotherapy, which is often associated with diminished ovarian function (Bines *et al*, 1996; Goodwin *et al*, 1999; Minton and Munster, 2002). Fertility information is a priority for young women with breast cancer but provision of such information is often inadequate (Partridge *et al*, 2004; Peate *et al*, 2009).

Following diagnosis of early breast cancer, women need to make rapid decisions regarding fertility interventions before commencing chemotherapy. Decision making about fertility preservation is complex with many ill-defined risk factors, such as fertility-treatment-induced increases in ovarian sex steroid levels, which are contraindicated in women with breast cancer (Oktay *et al*, 2005; Lee *et al*, 2006; Partridge *et al*, 2007). Further, fertility decisions usually need to be made shortly after a new cancer diagnosis, concurrent with cancer treatment decisions. Therefore, it is vital that clear and current information about fertility is provided in a timely manner.

The American Society for Clinical Oncology has recommended that fertility preservation be discussed early in women's treatment trajectory (Lee *et al*, 2006). It has been previously reported that fertility-related issues are often inadequately addressed in clinical management (Biglia *et al*, 2003; Partridge *et al*, 2004; Duffy *et al*, 2005; Thewes *et al*, 2005). Recent data has indicated that rates of fertility referrals are increasing in the United States (Lee *et al*, 2011), yet another study reported that <25% of US oncologists report referring patients for fertility preservation, only 38% reported knowledge of the American Society of Clinical Oncology guidelines, and <25% reported distributing fertility-related materials (Quinn *et al*, 2009). Furthermore, a study among Irish cancer specialists has reported that awareness of ART success rates was poor and a barrier to referrals (Collins *et al*, 2011). In Australia, there are no clear guidelines for referral despite the existence of public and private fertility clinics, and fertility treatment is not available without cost. The complexity of discussing fertility-related issues, including current relationship status, plans for children, and the range of fertility-preserving options available further hinder timely and adequate exchange of information (Duffy *et al*, 2005; Quinn *et al*, 2007, 2008). It is not surprising that young early breast cancer patients have unmet fertility-related information needs (Knobf, 2001; Thewes *et al*, 2003, 2004, 2005; Partridge *et al*, 2004).

Decision aids (DAs) are educational materials designed to assist with treatment decision-making, addressing individual values and preferences (Janis and Mann, 1977). They increase knowledge and reduce decisional conflict without increasing anxiety (O’Connor *et al*, 2009). The efficacy of a DA has not previously been evaluated in the context of fertility and early breast cancer, in particular the impact the DA may have on distress (anxiety and depression) and on other decision-making outcomes (such as, decisional conflict and regret, and informed choice). Anxiety and depression were assessed to measure whether receiving detailed, potentially distressing information about fertility would lead to adverse emotional outcomes.

The DA is a C5-sized booklet that contains information about breast cancer and female fertility, and a discussion of the different fertility options available. It also includes a series of values clarification exercises that list the advantages and disadvantages of each treatment option to be rated by participants as an option that they are either ‘leaning’ towards or against. Development and pilot testing of the fertility DA has been previously reported (Peate *et al*, 2011b) and the DA can be found on the Cancer Council of Australia's website (Peate *et al*, 2011a).

The current study prospectively evaluated the efficacy of this DA compared with usual care among young women diagnosed with early breast cancer. Specific aims were to:

Compare changes in decision-related outcomes, including decisional conflict about fertility treatment decisions (primary outcome) and knowledge, over time.
Compare decision-related outcomes, including decisional regret about treatment decisions, and informed choice at 1 and 12 months post diagnosis.
Examine potential changes in anxiety and depression as a result of the use of the DA compared to usual care.

## Methods

### Participants

Participants were consecutive early breast cancer patients (stage I, II, and III, excluding ductal carcinoma *in situ* and metastatic disease) referred to 1 of 19 oncology clinics around Australia between 2006 and 2009. Approval was obtained from all institutional review boards, and all women provided written informed consent. Inclusion criteria were: 18–40 years of age; diagnosed with invasive early breast cancer (as defined above); proficient in English; pre-menopausal at time of diagnosis; not yet having commenced adjuvant therapy; and interest in having a child. Eligible women were invited to participate by breast care nurses (BCNs) at each site within a week of their diagnosis and before any medical oncologist or fertility specialist appointments.

### Study design

The first 81 women recruited across all sites received a guide on early breast cancer developed for consumers, currently distributed to women at many clinics as part of usual care (National Breast Cancer Centre, 2003). This number was approached to account for attrition. The next 52 participants received the DA in addition to the consumer guide. A non-randomised trial design was undertaken to prevent participants sharing brochures.

### Procedure

Women were identified and invited to participate, soon after diagnosis, by the breast care nurses at each site, who were trained in the recruitment process. After consenting to participate, participants were given a package containing an invitation letter, baseline questionnaire, reply-paid envelope, and a sealed envelope with instructions not to open it until the questionnaire was completed. Upon completing the questionnaire, participants were asked to read the consumer guide (usual care control) or DA, which included completing the values clarification exercises (intervention), supplied in the sealed envelope before their oncologist appointment, if possible. Follow-up questionnaires were posted 1 and 12 months later. Participants were asked to complete paper-based self-administered questionnaires.

### Measures

#### Demographic, reproductive and disease-related data

Age at diagnosis, relationship status, parity, desire for more children, and highest level of education attained were recorded at baseline.

#### Intended decision about fertility treatment

Two items, measured at baseline and 1-month follow-up, asked ‘At this point in time, are you leaning towards…’ (a) ‘waiting to see if fertility returns after treatment’ and (b) ‘*in vitro* fertilisation’. Participants could opt to include intended decisions for ‘other’ techniques (not included in analyses due to limited data).

The following outcome measures were administered at all three time points:

*Decisional conflict scale (DCS)*: The 10-item low literacy version of this scale assessed decisional conflict regarding ‘different fertility treatments’ (O’Connor, 1995). Scores >37.5 on the overall scale (range 0–100) indicate high decisional conflict, which is characterised by decision delay and/or uncertainty about decision implementation (O’Connor, 1995).

*Knowledge of fertility-related information*: The items were selected based on previous research exploring the unmet fertility-related needs of this population (Thewes *et al*, 2005). Ten true-false items measured knowledge of (i) assisted reproductive technologies; (ii) the impact of different therapy regimens on fertility; and (iii) the impact of pregnancy after breast cancer on prognosis. A total knowledge score was calculated (range, 0–10). The scale had satisfactory internal consistency with Cronbach's alpha of 0.63.

*Hospital anxiety and depression scale*: The 14-item Hospital Anxiety and Depression Scale was used to measure emotional disturbance (Zigmond and Snaith, 1983; Ibbotson *et al*, 1994). It has two subscales measuring anxiety and depression, with each subscale ranging 0–21 (Zigmond and Snaith, 1983; Ibbotson *et al*, 1994).

The following outcome measures were administered at the two follow-up assessments only:

*Multidimensional measure of informed choice* (*MMIC*): This assessed whether women made treatment decisions with adequate knowledge (based on the Knowledge of Fertility-Related Information Scale) in accordance with their values, assessed with an adaptation of the five-item scale to assess attitudes towards fertility treatment in regard to feeling ‘beneficial’/‘harmful’, ‘important’/‘unimportant’, ‘a good thing’/‘a bad thing’, ‘pleasant’/‘unpleasant’, and ‘worthwhile’/‘not worthwhile’ (Michie *et al*, 2002). Response options ranged from ‘very positive’ (scored as 1) to ‘very negative’ (scored as 7). The MMIC was scored in accordance with the instructions provided by the developers – a total score was obtained by summing an individual's items (scores ranged from 5 to 35) (Michie *et al*, 2002). Two groups were classified as having made an informed choice: those who scored above the sample median on the knowledge of fertility-related information scale, had a positive attitude towards fertility treatments, and decided to undergo fertility treatments by the time of follow-up, and those who had a good knowledge score, a negative attitude towards fertility treatments, and did not undergo fertility treatments (Michie *et al*, 2002). All other women were categorised as having made an uninformed choice. The MMIC has good internal consistency (Cronbach's alpha of 0.66 and 0.81 for the knowledge and attitudes subscales, respectively, for this study) and excellent predictive and discriminant validity (Michie *et al*, 2002).

#### Decision regret scale (DRS) – fertility interventions

This five-item scale was used as a quantifier of health care decision regret (Brehaut *et al*, 2003) about fertility treatment decisions, with higher total scores indicating more regret (range 0–100).

*Use of materials:* One item determined whether, and the extent to which, materials were read.

*Satisfaction with, and helpfulness of, educational materials:* Three items elicited satisfaction with, and perceived helpfulness of, the educational materials.

*Actual decision:* Participants were asked about whether they received adjuvant treatments and fertility-preserving interventions.

*Partner's involvement*: Four items assessed the use of the educational materials by, and impact on any partners. These included whether the educational materials were shared with the partner; the extent to which partners had read the materials; whether the materials stimulated discussion between partners; how useful the partners considered the booklet; and whether the partner contributed to the decision-making process about fertility-related issues.

*Clinician discussion and referral*: Four items assessed frequency of contact, thoroughness of fertility-related discussions, and extent to which discussion and fertility referrals were prompted by study materials.

The following outcome measure was administered at the last follow-up assessment only:

*DRS – cancer treatments* (Brehaut *et al*, 2003): This *w*as used to assess regret about decisions made about cancer treatments. Higher total scores indicated greater regret (range 0–100).

### Statistical analyses

An intention to treat analysis was undertaken. Linear mixed effects models (with random baseline measurements and slopes) were used to examine the effect of the DA on outcome variables assessed across three occasions (DCS, knowledge, anxiety, and depression), adjusting for potential confounders and for curvilinear change (time^2^). Confounders (education, relationship status, parity, and desire for more children) were incorporated into a statistical model if they were theoretically relevant and resulted in a >20% change in the treatment effect estimates. Confounders were also included if they were associated with substantial reduction in Akaike's Information Criterion and Schwarz's Bayesian Information Criterion. Depression scores were log-transformed, given their skewed distributions. For variables collected at 1 and 12 months only, raw scores were used for analyses. Linear regression was used to assess differences between groups for continuous variables (DRS), and logistic regression was used to assess group differences for binary outcome variables (MMIC).

The target sample size was 64 participants in each group. This would provide sufficient power (80%) to detect a difference of 0.5 between groups (i.e., a medium effect size; Cohen, 1988) in the primary outcome variable (DCS). This has been considered clinically important in a range of cancer-related scenarios (Frost, 2002).

## Results

### Response rates

**Figure 1:**
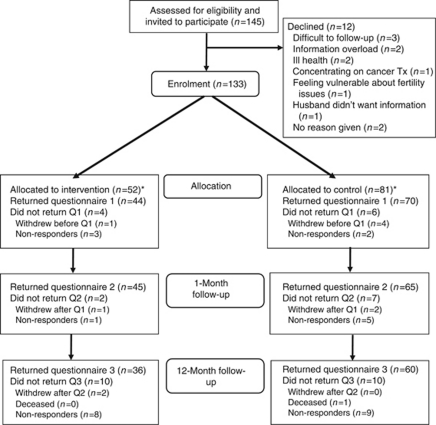
Participant flowchart. Q1, questionnaire one; Q2, questionnaire two; Tx, treatment; ^*^four participants who were allocated to the intervention and five participants to the control did not respond to any questionnaires and have not been included in the statistical anaylses.

A total of 145 eligible women were invited to participate (Figure 1), of these 70 women in the control group and 44 in the intervention group returned the baseline questionnaire. The final questionnaire was returned by 96 women (60 usual care and 36 intervention participants). The target sample size was not achieved as a result of slower than anticipated recruitment, and the trial was stopped before the target sample size in the DA group was achieved. Participants who completed at least one questionnaire (*N*=120, 72 and 48 participants allocated to usual care and the DA, respectively) were included in the analysis. This represented 83.3% of eligible subjects and 90.2% of those agreeing to participate. Due to slower than anticipated recruitment rates, the study was closed before reaching the target sample size; however, the sample size was large enough to detect differences.

### Participant characteristics

There were no significant differences in demographics between DA and usual care groups (Table 1). Most participants did not have children (69%, *N*=79). Of this group, 82% (*N*=65) reported that they planned to have children or that they were unsure whether they wished to have any children (18%, *N*=14). Of the 35 participants who already had children, 46% (*N*=16) definitely planned to have more children and the rest (54%, *N*=19) were unsure. Overall, most participants reported that they planned to have (more) children (71%, *N*=81).

Unadjusted mean scores and percentages are presented for all outcomes separately for the DA and usual care groups in Table 2.

The majority of participants (96%) who received the DA reported reading the materials, compared with 83% of participants allocated to usual care. The extent to which this was read is shown in Table 3.

### Decisional conflict scale

**Figure 2:**
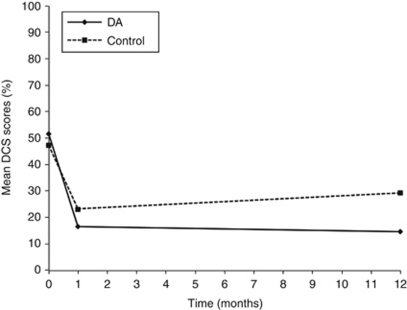
Mean DCS scores over the three data collection points.

Those who received the DA had a greater reduction in decisional conflict than those who received usual care over 12 months (Figure 2). Using a mixed effects model with random baseline measurements, the effect of the DA was significant after adjustment for education, desire for more children, and uncertainty at baseline (treatment group by time interaction: *β*=−1.51; 95%CI: −2.54 to 0.48; *P*=0.004), in a curvilinear manner (*β*=2.42; 95%CI: 1.88–2.96; *P*<0.001). Adjusted results identified a negligible mean difference of 1.46 in DCS between groups at 1 month (s.e.=3.66; 95%CI: 5.77–8.69; *P*=1.00). Decisional conflict scale scores were 15.30 units lower in the DA group at 12 months (s.e.=5.52; 95%CI: −26.17 to 4.43; *P*=0.02).

### Knowledge of fertility-related information

**Figure 3:**
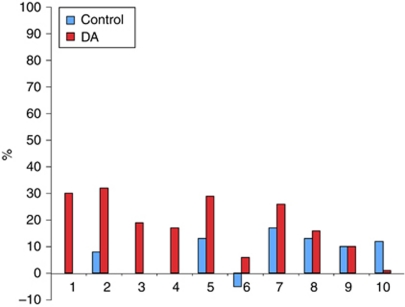
Change in the percentage of correct answers in the knowledge scale from baseline to 12 months, shown in order of the greatest difference between groups. Key: (1) IVF (*in vitro* fertilisation) has highest success rate of fertility options (True); (2) some fertility procedures are still experimental and not widely available (true); (3) pregnancy after breast cancer treatment is safe for mother and baby (true); (4) impact of chemotherapy on fertility is not dependent on age (false); (5) IVF will not delay cancer treatment (false); (6) pregnancy after breast cancer treatment will increase chance of recurrence (false); (7) hormonal therapy will not cause infertility except for time spent on treatment (true); (8) many breast cancers depend on hormones to grow – thus some fertility drugs are not recommended (true); (9) fertility treatment can be costly (true); (10) chemotherapy impacts on fertility by depleting eggs in the ovaries (true).

Figure 3 shows the change in correctly answered questions from baseline to 12 months in both study arms, and shows that across most items there was an increase in correct responses from baseline to final follow-up.

**Figure 4:**
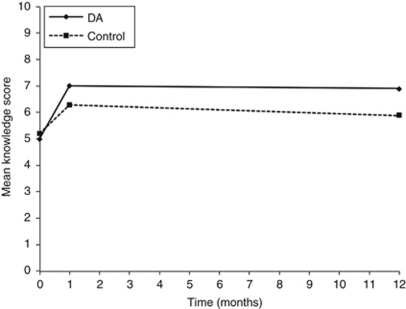
Mean knowledge scale scores over the three data collection points.

Use of the DA significantly improved fertility-related knowledge (Figure 4). Using a random baseline and slopes mixed effects model with adjustment for education, desire for more children and uncertainty about the decision at baseline, a significant improvement in knowledge was noted (treatment group by time interaction: *β*=0.09; 95%CI: 0.01–0.16; *P*=0.02) in participants who received the DA, compared with the usual care group. Adjusted results identified a negligible mean difference of 0.01 in knowledge scores between groups at 1 month (s.e.=0.032; 95%CI: −0.65 to 0.64; *P*=0.99). Knowledge scores were 0.94 units lower in the DA group at 12-month follow-up (s.e.=0.44; 95%CI: 0.08–1.81; *P*=0.03).

### Multidimensional measure for informed choice

There were no significant differences in the proportions of participants who made an informed *vs* an uninformed choice about fertility based on group allocation at 1 month (OR=2.12; 95%CI: 0.32–2.26; *P*=0.75), and at 12-month follow-up (OR=4.41; 95%CI: 0.59–3.75; *P*=0.10), after adjusting for relationship status, education, parity, and desire for more children.

### Hospital anxiety and depression scale

There was no significant difference in anxiety between treatment groups after adjusting for education, parity, and desire for more children (treatment group by time interaction: *β*=0.02; s.e., 0.06; 95%CI: −0.10 to 0.14; *P*=0.73) or depression (treatment group by time interaction: *β*=0.09; 95%CI: −0.03 to 0.21; *P*=0.14, adjustment unnecessary).

### Decisional regret scale

At 1 month, DRS scores regarding fertility-related decisions were not significantly different between groups (*β*=4.75; 95%CI: −2.7 to 12.22; *P*=0.21), or after adjusting for education (*β*=5.40; 95%CI: −2.19 to 12.99; *P*=0.16). After adjusting for education, participants who received the DA had significantly lower decisional regret at 12 months (*β*=−3.73; 95%CI: −7.12 to −0.35; *P*=0.031).

### Satisfaction with information

Sixty-seven percent of participants receiving usual care and 70.5% of participants allocated the DA were satisfied with the information provided on the impact of breast cancer treatment on fertility. Similarly, 54.4% and 77.3% were satisfied with the information given on the impact of treatment and fertility options, respectively. Compared with participants who received usual care, participants allocated the DA were significantly more satisfied with the materials they received on the impact of breast cancer treatment on fertility (*χ*^*2*^=15.49; *P*<0.001) and on the different fertility options available (*χ*^2^=10.66; *P*=0.005). Participants were also significantly more likely to score the DA more highly on a helpfulness scale in terms of making fertility-related decisions (*χ*^2^=0.61; *P*=0.002).

### Partner's involvement

The impact of the DA on partner's involvement in reading and discussing the educational materials and contributing to fertility-related decision making is shown in Table 4. Partner's involvement did not significantly differ between groups. Partners were considered to be ‘very’ or ‘extremely’ involved in the decision-making process (77.6%) with most participants rating the DA as ‘somewhat’ or ‘very’ useful to partners (74.2%).

### Fertility-related discussions with oncologists

Perceived thoroughness of discussions with oncologists and the extent to which the discussions were prompted by the educational materials are reported in Table 5. Most women had discussed fertility to some extent with their medical oncologists (92.5% of controls and 97.7% of women who received the DA, *χ*^2^=1.50; *P*=0.47).

### Referrals to fertility specialists

Sixty-two percent of participants who received the DA and 55.6% allocated to usual care were referred to a fertility specialist (*χ*^*2*^=0.48; *P*=0.49). One participant in each group sought a fertility specialist, although these patients were not referred by their oncologists. Of those who received a referral to a fertility specialist, 91.4% of controls consulted or were planning to see a fertility specialist, compared with 97.7% participants who received the DA (*χ*^*2*^=0.71; *P*=0.40).

### Intended decisions and fertility treatment uptake

Leaning towards the ‘wait and see’ option did not change significantly from baseline to 1 month between groups (*P*=0.31). Additionally, no differences in change scores were observed for intention to undergo IVF between groups (*P*=0.42). For the total patient sample, 79.7% of controls reported having made a decision about fertility treatment at 1 month compared with 91.1% of participants who were allocated the DA (*χ*^2^=2.62; *P*=0.11). At 12 months, 71.2% of participants allocated to usual care and 76.5% of participants allocated to the DA had made a decision about fertility preserving options (*χ*^2^=0.84; *P*=0.36).

## Discussion

This study is the first to report the impact of a fertility DA on decisional conflict, decision regret, and fertility choices in young breast cancer patients. The DA reduced decisional conflict about fertility-related treatment options and reduced decisional regret about fertility treatments compared with usual care over 12 months. The DA also improved fertility-related knowledge. The reduction in decisional conflict and improvements in knowledge may be considered clinically important (Ringash *et al*, 2007). It is likely that the DA achieved these outcomes by providing more comprehensive information about fertility options and by facilitating timely and personalised decision making (O’Connor *et al*, 1998). Lower decisional regret indicates reduced distress or remorse after a decision (Brehaut *et al*, 2003). Ultimately, these outcomes indicate that access to the DA increased satisfaction with choices made and enabled women to feel more informed about their options and clearer regarding their personal values (O’Connor *et al*, 2009).

Access to the DA did not impact on uptake of fertility-preserving interventions. Uptake of fertility interventions is not only dependent on information, but also on personal circumstances (such as having a male partner), availability and cost of interventions, previous fertility as well as religious and personal beliefs (Lee *et al*, 2006; Dutney, 2007; Jukkala, 2009; Ata and Seli, 2010; Eisenberg *et al*, 2010; Hershberger and Pierce, 2010). In addition, uptake of interventions is likely to be influenced by clinician recommendations (Young, 1996a, 1996b; Cyrus-David and Strom, 2001; Bober *et al*, 2004; Taylor and Taguchi, 2005; McGregor *et al*, 2007). Little is known about how and why women and their partners make decisions about fertility interventions following a cancer diagnosis, or how clinicians decide who to refer to a fertility specialist. In particular, the relative influence of patient wishes and clinical recommendation is poorly understood. It is likely that patients are highly influenced by clinical recommendations regarding fertility interventions, particularly from their medical oncologist (Silvestri *et al*, 2003; Lam *et al*, 2005). Thus, it is important to provide cancer clinicians with accurate information about fertility options so that patients are advised and referred appropriately. Improved understanding of how oncologists make decisions about fertility specialist referral is needed.

Reported discussions with oncologists and referrals to specialists did not differ, with or without the DA. Our findings suggested that most medical oncologists discussed fertility with breast cancer patients of reproductive age. These statistics are higher than the rate of ∼70% reported in previous studies (Partridge *et al*, 2004; Thewes *et al*, 2005), and may reflect selection bias in clinicians who participated in our study. A national survey of American oncologists reported that those with favourable attitudes towards fertility preservation were nearly twice as likely to discuss the issue, compared with those who had unfavourable attitudes, with male oncologists approximately half as likely to refer patients as their female colleagues (Quinn *et al*, 2009).

Compared with participants who received usual care, those with access to the DA reported higher rates of satisfaction with the fertility information received (>70%), which is above the 51% previously reported as having their concerns addressed adequately (Partridge *et al*, 2004). Limited discussion may be the result of women or their clinicians feeling uncomfortable about the topic, particularly as the primary concern for most is the cancer diagnosis and treatment. Younger breast cancer patients may expect that their health care providers, as the experts, will discuss all that is relevant without prompting, in which case the opinion of the oncologist regarding fertility may be crucial. Fertility treatments are rapidly evolving. The level of current knowledge of oncologists about potential fertility preserving options remains unknown. All participants in this study were considering pregnancy in the future, but only half were referred to a fertility specialist, who could offer fertility-preservation techniques. This is greater than the reported rate of >25% referrals to fertility specialists reported in a 2009 study of a random sample of oncologists in the United States (Quinn *et al*, 2009). Again, this may reflect either a selection bias of participating oncologists, or a genuine increase in fertility awareness among oncologists. The same study reported that <25% of clinicians distributed fertility preservation materials (Quinn *et al*, 2009). In comparison, all participants in our study were given fertility information, either the control booklet or DA, which may have been the impetus for a higher referral rate. Though clinic staff was trained in recruitment procedure to minimise variation across sites, we acknowledge that there is the potential that different clinicians’ attitudes may influence decision making. The numbers recruited at each site for this study were limited; thus, we lacked the capacity to compare individual effects. More information is needed about how breast cancer specialists approach fertility issues and how the latest information about fertility can be effectively provided to oncologists, and subsequently delivered to patients (including involving patients in research).

In addition to the limitations discussed above, the non-randomised design of the study may lead to bias. However, studies in similar populations using this study design have not shown bias as a result of time (Goodwin *et al*, 1999). Additionally, the target sample size was not achieved most likely a result of slower than anticipated recruitment as a consequence of strict eligibility criteria (age, desire for future fertility, and not yet started adjuvant treatment). It was possible that some women who were eligible for study participation were not recruited in the short time between diagnosis and commencing treatment. There is also the potential that low numbers were a result of the fact that women were approached at a highly stressful time. An increased sample may have improved the power to detect a difference where trends were seen (MMIC). Nevertheless, the sample size obtained appeared large enough to detect a statistically significant result in the primary outcome variable, DCS. This may have been because the actual treatment effect was greater than originally anticipated.

Our results raise some interesting questions that may need further exploration. Most of our participants reported that their partners were involved in the fertility treatment decision-making process, yet very little is known about how partners perceive their role despite fertility-related decisions being a decision for both parties. Understanding the mechanisms of fertility-related decision making should incorporate this factor.

Within individual items of our knowledge scale there were improvements of varying extent. Further exploration of the specific informational domains would assist in improving the information delivered and improve the DA. Other considerations include that the DA will require regular updating as reproductive technologies advance, bringing ongoing costs. Online resources may facilitate updating and reduce costs. Also, the DA is currently available only in English and is not appropriate for culturally and linguistically diverse populations. Further research into these issues would further advance support this body of work.

## Conclusion

We have shown that this fertility DA reduced decisional conflict and regret about fertility-related treatment options in young breast cancer patients. Lower decisional conflict and regret indicate increased satisfaction with choices with respect to personal values. The DA also improved fertility-related knowledge, which is an important aspect of informed decision making. The DA also improved satisfaction with information received and was considered helpful by patients. These findings suggest that this DA should be widely distributed to young breast cancer patients considering future pregnancies before chemotherapy.

## Figures and Tables

**Table 1:** Demographic characteristics (*N*=120)

	**Control**		**Intervention**		**Total**	
**Variable**	***N***	**Mean (s.d.)**	***N***	**Mean (s.d.)**	***N***	**Mean (s.d.)**
Age in years	72	33.8 (4.0)	48	32.3 (4.7)	120	33.23 (4.3)
Relationship length in yearsa,b	23	4.6 (3.9)	18	4.4 (3.3)	41	4.49 (3.6)
						
		%		%		%
*Relationship status* a						
Committed relationship	53	74.6	36	76.6	89	75.4
Not in a committed relationship	18	25.4	11	23.4	29	24.6
						
*Parity*						
No	46	63.8	36	75.0	79	69.3
Yes	26	36.1	12	25.0	35	30.7
						
*Highest level of education*						
High school only	13	18.3	5	10.6	18	15.3
Certificate/diploma	18	25.4	8	17.0	26	22.0
Undergraduate degree	27	38.0	20	42.6	47	39.8
Postgraduate degree	13	18.3	14	29.8	27	22.9
						
*Medical or allied health training*						
No	59	83.1	39	83.0	98	83.1
Yes	12	16.9	8	17.0	20	16.9
						
*Stage*						
Stage I	7	12.1	3	7.0	10	9.9
Stage II	18	31.0	15	34.9	33	32.7
Stage III	33	56.9	25	58.1	58	57.4

a Several participants did not provide information for this item.

b Median=3.0, inter-quartile range: 2.0–5.5 years.

**Table 2:** Dependent variable mean scores and proportions on the DCS, HADS, DRS, and multidimensional measure of informed choice scale

	**Control**		**Intervention**		**Unadjusted analysis^a^**	
	***N***	**Mean (s.d.)**	***N***	**Mean (s.d.)**	***β***	***P*-value**
*Knowledge score*						
T0	69	5.2 (2.13)	44	5.0 (2.61)	0.08	0.03
T1	65	6.3 (2.28)	44	7.0 (2.16)		
T2	59	5.9 (1.92)	33	6.9 (2.01)		
						
*DCS*						
T0	70	47.2 (30.98)	44	51.6 (32.76)	1.43	0.007
T1	64	23.1 (30.56)	45	16.6 (26.48)		
T2	58	29.3 (30.96)	34	14.7 (23.74)		
						
*HADS: depression scale*						
T0	70	5.1 (3.71)	44	4.6 (4.72)	0.02	0.15
T1	65	5.0 (3.38)	45	5.3 (4.68)		
T2	59	3.2 (3.18)	33	4.0 (4.94)		
						
*HADS: anxiety scale*						
T0	70	8.9 (4.32)	44	8.5 (4.40)	0.04	0.56
T1	65	7.8 (3.71)	45	7.3 (4.92)		
T2	59	7.0 (4.52)	33	7.1 (4.27)		
						
*DRS: fertility*						
T1	61	19.7 (18.86)	43	24.4 (19.00)	4.75	0.21
T2	57	49.1 (8.24)	32	45.8 (8.97)	3.34	0.047
						
*DRS: cancer*						
T2	58	47.8 (7.79)	34	46.5 (7.23)	−0.08	0.43
						
**Proportional data for dichotomous variables (%)**					**OR**	
*Multidimensional measure of informed choice*						
T1	37	60.7% Informed	22	64.3% Informed	3.22	0.071^b^
T2	29	51.8% Informed	20	64.5% Informed	5.44	0.25^b^
						
					***χ* ^2^**	
*Actual fertility treatment* a						
*In vitro* fertilisation						
T2	53	24.5%	33	30.3%	0.63	0.43
‘Wait and see’						
T2	57	61.4%	33	66.6%	0.78	0.38
						
*Actual cancer treatment*						
Radiotherapy						
T2	59	78.0%	35	77.1%	0.33	0.87
Chemotherapy						
T2	59	84.7%	35	82.9%	0.15	0.70
Endocrine therapy						
T2	59	66.1%	34	70.6%	0.58	0.45

Abbreviations: DCS=decisional conflict scale; DRS=decisional regret scale; HADS=hospital anxiety and depression scale; s.d.=standard deviation; T0=baseline; T1=1 month; T2=12 months.

a Using a mixed effects model with random baseline measurements, this value represents treatment group by time interaction, unadjusted for confounders.

b Using a linear or logistic regression models, unadjusted for confounders.

**Table 3:** Extent to which information materials given as part of this study were read

	**Control (%)**	**DA (%)**
Not at all	17	5
Yes, briefly	8	14
Yes, just the parts I felt were relevant	22	25
Yes, quite thoroughly	34	27
Yes, from cover to cover	19	29

Abbreviation: DA=decision aid.

**Table 4:** Partner involvement in sharing of materials, discussion, and decision making

		**Control**		**Intervention**			
**Item**	**Responses**	***N***	***%***	***N***	***%***	***χ2***	***P*-value**
Materials read by partners	Yes	33	67.3	17	51.2	2.08	0.15
	No	16	32.7	16	48.5		
							
Thoroughness in which partner read the information^a^	Briefly	9	29.4	6	33.3	0.91	0.34
	Just relevant parts	8	23.5	6	33.3		
	Quite thoroughly	13	38.2	6	33.3		
	Cover to cover	3	8.8	0	0.0		
							
Discussions stimulated by the materials	Yes	17	36.2	12	38.7	0.052	0.82
	No	30	63.8	19	61.3		
							
Perceived usefulness of the material for the partner^b^	Not at all	2	5.3	3	10.7	0.48	0.49
	Not very	9	23.7	3	10.7		
	Somewhat	16	42.1	14	50.0		
	Very useful	11	28.9	8	28.6		
							
Partner contribution to fertility-related decision making^c^	Yes	43	87.8	30	93.8	—	—
	No	6	12.2	2	6.2		
							
Perceived level of partner involvement in fertility-related treatment decisions^d^	Not at all	0	0.0	0	0.0	0.042	0.84
	A little	4	9.3	4	13.3		
	Quite	4	9.3	5	16.7		
	Very	13	30.2	7	23.3		
	Extremely	22	51.2	14	46.7		

a Due to small cell sizes, for *χ*^2^ analyses responses were grouped into ‘briefly and just relevant parts’ and ‘quite thoroughly and cover to cover’.

b Due to small cell sizes, for *χ*^2^ analyses responses were grouped into ‘not at all and not very’ and ‘somewhat and very useful’.

c Due to small cell sizes statistical tests could not be conducted.

d Due to small cell sizes, for *χ*^2^ analyses responses were grouped into ‘not at all, a little and quite’ and ‘very and extremely’.

**Table 5:** Reported details about discussions with medical oncologists about fertility related issues

		**Control**		**Intervention**			
**Item**	**Responses**	***N***	***%***	***N***	***%***	***χ2***	***P*-value**
Thoroughness of fertility-related discussions with oncologist^a^	Not at all	4	6.2	1	2.3	1.50	0.47
	Briefly	18	27.7	19	43.2		
	Moderately	22	33.8	12	27.3		
	Quite a bit	13	20.0	9	20.5		
	Extensively	8	12.3	3	6.8		
							
Who was the discussion initiated by?^b^	Patient	25	38.5	16	37.2	<0.001	1.00
	Clinician	36	55.4	23	53.5		
	Not discussed	4	6.2	4	9.3		
							
Extent to which discussion was prompted by educational materials^a^	Not at all	28	43.1	12	26.7	1.28	0.53
	A little bit	12	18.5	11	24.4		
	Moderately	16	24.6	15	33.3		
	Quite a bit	7	10.8	4	8.9		
	Very much	2	3.1	3	6.7		

a Due to small cell sizes, for *χ*^2^ analyses responses were grouped into ‘not at all and briefly’, ‘moderately’, and ‘quite a bit and extensively’.

b *χ*^2^ analyses excluded those in which fertility was not discussed.
